# The fecal microbiota of healthy donor horses and geriatric recipients undergoing fecal microbial transplantation for the treatment of diarrhea

**DOI:** 10.1371/journal.pone.0230148

**Published:** 2020-03-10

**Authors:** Caroline A. McKinney, Bruno C. M. Oliveira, Daniela Bedenice, Mary-Rose Paradis, Melissa Mazan, Sophie Sage, Alfredo Sanchez, Giovanni Widmer

**Affiliations:** 1 Department of Clinical Sciences, Cummings School of Veterinary Medicine at Tufts University, North Grafton, MA, United States of America; 2 Department of Infectious Diseases and Global Health, Cummings School of Veterinary Medicine at Tufts University, North Grafton, MA, United States of America; 3 Faculdade de Medicina Veterinária, Universidade Estadual Paulista (Unesp), Araçatuba, Brazil; Washington State University - Spokane, UNITED STATES

## Abstract

**Background and aims:**

Fecal microbial transplantation (FMT), a treatment for certain gastrointestinal conditions associated with dysbiosis in people, is also empirically employed in horses with colitis. This study used microbiota high-throughput sequencing to compare the fecal microbial profile of healthy horses to that of geriatric microbial transplant recipients experiencing diarrhea and tested whether FMT restores microbiota diversity.

**Methods:**

To evaluate the effect of environment and donor characteristics on the intestinal microbiota, fecal samples were collected per rectum from 15 healthy young-adult (2–12 years) and 15 geriatric (≥20 years) horses. Additionally, FMT was performed for 3 consecutive days in 5 geriatric horses with diarrhea using feces from the same healthy donor. Fecal samples were collected from both donor and recipient prior to each FMT and from recipients 24 hours following the last FMT. The profile of the fecal bacterial microbiota was compared using 16S amplicon sequencing.

**Results and conclusions:**

In contrast to diet and farm location, age did not significantly affect the healthy equine fecal microbiota, indicating that both healthy geriatric and young-adult horses may serve as FMT donors. The fecal microbiota of horses with diarrhea was significantly more variable in terms of β-diversity than that of healthy horses. An inverse correlation between diarrhea score and relative abundance of Verrucomicrobia was identified in surviving FMT recipients. At study completion, the fecal microbiota of horses which responded to FMT had a higher α-diversity than prior to treatment and was phylogenetically more similar to that of the donor.

## Introduction

Intestinal microbiome imbalance, or dysbiosis, is a key factor in the development of diarrhea and equine colitis, which remains a leading cause of critical illness in horses, with an estimated disease fatality of 25.4% to 35%.[1, 2] Antibiotic therapy is often ineffectual and even worsens diarrheal disease by destroying beneficial commensal bacteria in the gut and allowing pathogenic species to expand. A lack of specific and effective therapies for equine colitis prevents clinicians from rapidly reversing disease-associated fluid losses and systemic inflammation that increase complication rates and result in the need for intensive care throughout prolonged hospital stays. Early therapeutic intervention is, therefore, necessary to circumvent potentially life-threatening complications of colitis (such as severe fluid loss, laminitis, coagulopathy, cardiovascular dysfunction) and improve overall outcome. The specific cause of colitis remains unknown in more than 50% of cases; in addition to bacterial and viral infections, disruption of the delicate microbial balance of the gut (dysbiosis) may result in colitis and may further diminish resistance to enteric pathogens.[3] Emerging data suggest that fecal microbial transplantation (FMT) may be efficacious in restoring normalcy to the gut.[4] FMT constitutes the transfer of a suspension of fecal microorganisms from a healthy donor horse into the intestinal tract of the recipient horse in order to treat a specific disease associated with intestinal microbiome imbalance.[5] It may thus represent a novel, cost-effective therapy to successfully restore gut function in horses with colitis and diarrhea.

It has recently been shown that the fecal bacterial microbiota of horses with colitis of various etiologies is less diverse than that of unaffected animals.[3, 6] As with horses, acute and chronic diarrhea in humans may also be associated with intestinal dysbiosis. Consistent with the importance of a balanced intestinal microbiota, antibiotic therapy has been inadequate in the treatment of human colitis due to specific pathogens such as *Clostridium difficile*. A well-described clinical trial in humans with refractory, chronic *C*. *difficile* diarrhea showed over 85% effectiveness for one FMT, and over 90% effectiveness for two FMTs in comparison to standard treatment with vancomycin. A recent study in mice further demonstrated that autologous FMT significantly enhanced fecal and gastrointestinal microbiota reconstitution in mice following antimicrobial treatment.[7] Similarly, anecdotal information arising from a small number of horses suggests that fecal consistency normalizes following FMT[8] and that chronic diarrhea may improve in response to microbial transplantation.[9] Development of FMT as an inexpensive, effective means of restoring the colonic microbiota and thereby combating equine colitis would be of benefit to both the veterinary and equine industry. By leveraging the protective properties of the normal gut microbiota, this treatment modality may also counteract the ever-increasing problem of antibiotic-resistant microbial pathogens.

A recent study showed that the outcome of colitis is less favorable in geriatric compared to younger horses.[10] At the same time, the population of geriatric horses in North America has steadily increased from 5.6% in 1998 to 11.4% in 2015 (APHIS),[11] with a nearly 6-fold increase in geriatric horse admissions over a period of 10 years at the authors’ referral institution.[12] While equine geriatric medicine has, therefore, gained rising importance in the horse and veterinary industry, a consensus on how aging impacts the equine gut microbiota and the horse’s recovery from conditions associated with dysbiosis has not been reached. To date, conflicting associations between aging and microbiota diversity are reported.[13, 14] The current study therefore investigates both the eligibility of geriatric horses as FMT donors and the response of geriatric horses to FMT treatment.

Although recent studies have improved our understanding of the equine gut microbiota,[15, 16] little is known about the effect of FMT on either the recipient’s fecal microbiota or mechanisms leading to improved clinical outcome. The current study, conducted within ethical constraints governing the use of client horses, was designed to identify desirable FMT donor characteristics based on the impact of age, diet, and housing on the normal equine fecal microbiota, and to explore the short-term effects of FMT on microbiota diversity in geriatric horses with colitis.

## Materials and methods

This study was approved by the Cummings School of Veterinary Medicine Institutional Animal Care and Use Committee (#G2016-179) and Clinical Studies Review Committee (#161215) and met all requirements for ethical care and treatment of animals.

### Healthy age-matched control horses

Thirty clinically healthy horses of two age groups (15 young-adults [2–13 years old]; 15 geriatric horses [20–30 years old]) were selected from 5 housing facilities located in Massachusetts and Connecticut, based on the availability of phenotypically matched young and aged horses at each location, exposure to a comparable diet, and similar management. The latter selection criteria were used to determine which management and phenotypic features may contribute to differences in the fecal microbiota of healthy horses and should thus be considered in selecting a donor for fecal microbial transplantation. Prior to fecal collection, a complete diet history, medical history, and physical examination were obtained to ensure clinical health. Exclusion criteria included any recent gastrointestinal illness (colic, diarrhea), transport, medical treatment, or dietary supplementation with probiotics. Breed, age, body condition score, heart rate, respiratory rate, rectal temperature, attitude, and borborygmi were recorded prior to manure collection. Up to 10-mL of feces were collected per rectum using a clean gloved hand at two separate time-points 2 weeks apart and stored at -80°C for subsequent analysis. The experimental design and methods were approved by the Clinical Studies Review Committee and informed client consent was obtained for all horses.

A quantitative fecal egg count and Equine Diarrhea PCR panel for Coronavirus, *Clostridium difficile* toxins A and B, *Clostridium perfringens* antigens, *Lawsonia intracellularis*, *Neorickettsia risticii* and *Salmonella* sp. (Equine Diarrhea Panel, Research and Diagnostic Core Facility, University of California, Davis) were confirmed to be negative for 3 control horses that served as FMT donors to clinically ill geriatric patients with diarrhea. Prior to each FMT on 3 consecutive days, a 10-mL fecal sample was obtained from the donor manure pile that was subsequently processed and used as transfaunate for a single patient. All donor samples obtained on days 1–3 of transfaunation were stored and sequenced individually, thus analyzing a total of 3 FMT samples per donor obtained on consecutive days and administered to a single recipient.

### Geriatric colitis horses

Five geriatric horses hospitalized at Tufts Cummings School of Veterinary Medicine with diarrhea (pudding to watery consistency) served as fecal transplant recipients. Exclusion criteria included a history of gastrointestinal reflux within 3 days prior to enrollment in the study. The following clinical data were obtained: signalment; presenting complaint; predisposing factors such as antibiotic or non-steroidal anti-inflammatory therapy, recent long-distance travel, anesthesia, feed changes, or prior enteral treatment with mineral oil, surfactants, or cathartics; duration of diarrhea; development of complications such as laminitis or thrombophlebitis; length of hospitalization; and outcome. Frequency of diarrhea during hospitalization was recorded every 6 hours in clinical patients (colitis horses), and diarrhea quality described every 6 hours on a scale of 0–5 according to the following guidelines: 0—Normal, firm but moist balls of manure; 1 –Soft-formed balls of manure that lose their form upon reaching the ground; 2—Pudding-consistency manure that still holds some shape; 3—Pudding-consistency manure that spreads out upon reaching the ground; 4—Watery manure with some formed pieces; 5—Watery manure without formed pieces.

### Clinical procedures

Five client-owned geriatric horses (≥ 20 years old) that presented for colitis, or developed diarrhea in-hospital for any reason, received fecal microbial transplants from a single donor horse on 3 consecutive days. FMT was performed using standard clinical techniques. In short, 2.5 pounds of fresh manure was collected and mixed in 4 liters of lukewarm water within a bouffant cap (serving as a standard sieve; McKesson 24-inch Disposable Bouffant Caps) for 10 minutes. The mixture was subsequently strained and administered within 15 minutes of processing via nasogastric tube to the recipient horse. Horses were checked for reflux using no more than 2 L of water prior to administration of FMT, with <2 L net reflux considered acceptable. A 10 mL fecal sample was collected per rectum from each recipient prior to each FMT (days 1–3) and 24 hours following the last FMT (day 4) (S1 Table). All samples were stored at -80°C.

Clinical data are presented descriptively as mean +/- standard deviation (SD) or median +/- interquartile range (IQR) or range. Univariate statistical analyses were based on the normality of data distribution (Shapiro-Wilk test), employing independent samples T-test, Mann-Whitney U and Chi-Square analyses to compare data between geriatric and young-adult donor horses, with an accepted significance level of P<0.05. These numerical analyses were performed with the IBM SPSS Statistics 22 package.

### Microbiota analysis

The procedures for DNA extraction, amplicon library construction and bioinformatics were previously described [17–19]. Briefly, fecal DNA was PCR amplified to prepare amplicons of the V1V2 variable region of the bacterial 16S rRNA gene [20, 21]. The multiplexed amplicon library was size-selected on a Pippin HT system (Sage BioScience, Beverly, Massachusetts) and sequenced in an Illumina MiSeq sequencer at the Tufts University genomics core facility (tucf.org) using the single-end 300-nucleotide strategy. To control for technical variation introduced during PCR, library preparation and sequencing, the 16S library included two replicates of a randomly selected sample. Replication involved the separate processing of 2 subsamples of a sample and tagging each amplicon with a different 6-nucleotide barcode.

### Bioinformatics

FASTQ formatted sequences were processed using programs found in *mothur* [22] essentially as described [17]. Briefly, random subsamples of 5000 sequences per sample were processed. Pairwise weighted UniFrac phylogenetic distances [23] between samples were calculated in *mothur* using program *unifrac*.*weighted*. The UniFrac distance ranges from 0 for identical samples to 1 for samples that are completely different and share no sequences or no taxa. GenAlEx [24] was used to draw Principal Coordinate Analysis (PCoA) plots using weighted UniFrac distance matrices as input. Analysis of Similarity (ANOSIM) [25] was used to test the significance of clustering by treatment. Program *anosim* was run in *mothur* using weighted UniFrac distance matrices as input. Operational Taxonomic Units (OTUs) were obtained using program *cluster* and the OptiClust clustering method [26]. A distance cut-off of 3% was applied. Linear Discriminant Analysis (LDA) as implemented in program LEfSe [27] was used to identify statistically significant differences in OTU abundance profiles between two groups of samples defined by dietary treatment. Shannon diversity was also calculated in *mothur*. Constrained ordination analysis was performed in CANOCO [28].

**Sequence data accession numbers.** 16S sequence data were deposited in the Sequence Read Archive under study accession number PRJEB32490.

## Results

### Healthy age-matched control horses

Fecal samples were obtained from 15 paired young-adult (median age: 6.7 ± 3.7 years) and geriatric horses (median age: 22.6 ± 1.8 years) exposed to comparable management conditions, including 21 mares and 9 geldings. Aged and young-adult horses were matched based on phenotype, body condition score, sex and diet, except for one horse pair where a young gelding was matched with an aged mare (S2 Table). The most common breeds included Morgans (10/30; 33.3%), Thoroughbreds (8/30; 26.7%) and Western Breed Horses (8/30, 26.7%). Neither baseline physical examination parameters nor body condition score differed significantly between age-groups (P>0.05). All horses were fed a median of 2.25% ± 0.25 body weight first cut hay per day, except for one young–geriatric horse pair which received 2% body weight second cut hay per day. The amount of concentrate fed and the duration of pasture turnout did not differ between young and aged horses (P >0.05) (S2 Table).

### Geriatric colitis horses

Five geriatric (≥ 20 years of age) client-owned horses, including 3 geldings and 2 mares, presented for colitis or developed diarrhea while hospitalized, prior to receiving 3 consecutive daily FMT from a single donor. Duration of hospitalization ranged between 5 to 8 days, with 3/5 horses surviving to discharge. The horses’ historical, clinical and hematologic characteristics are specified in S3–S5 Tables. All horses underwent Salmonella Polymerase Chain Reaction (PCR) testing (3 samples collected at ≥12 hour intervals), while a combined equine diarrhea PCR panel was only performed in 4 of 5 horses with diarrhea (Equine Diarrhea Panel, Research and Diagnostic Core Facility, University of California, Davis: Coronavirus; Clostridium difficile toxins A and B; Clostridium perfringens (CP) antigen, CP alpha toxin, CP beta toxin, CP beta2 toxin, CP cytotoxin (netF), CP enterotoxin; Lawsonia intracellularis; Neorickettsia risticii and Salmonella) to identify potential etiologies of their colitis. Although one horse (F) tested positive for *Clostridium perfringens* antigen, all horses likely experienced undifferentiated colitis. Horse H did not have a diarrhea PCR panel performed but showed evidence of sand enteropathy based on abdominal radiographs and removal of enteric sand on rectal palpation.

### Microbiota analysis

**Fig 1:**
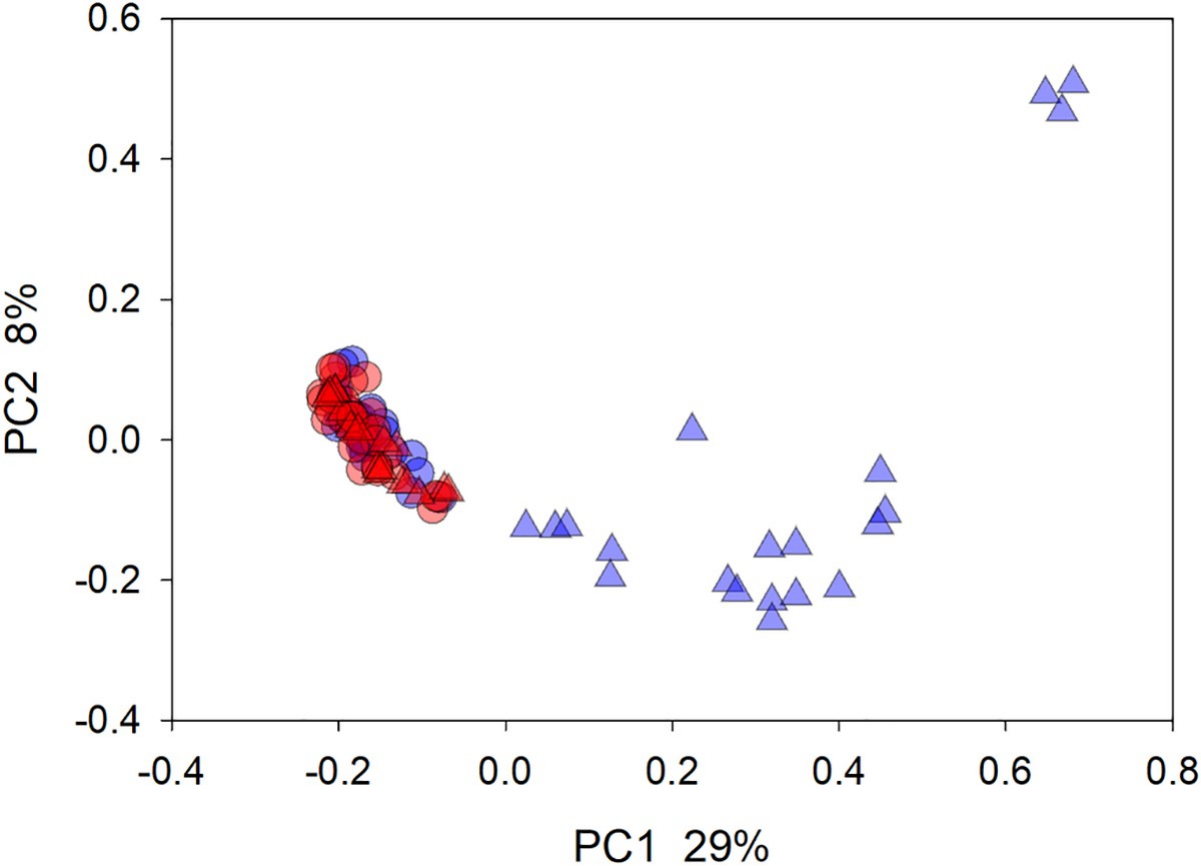
Principal coordinate analysis of all horses and all samples. The plot is based on weighted pairwise UniFrac distances between 97 samples. The distance between points is an approximate representation of the phylogenetic distance between 2 microbiota samples. Circles, 60 samples: 2 from each healthy old (n = 15) and from each young (n = 15) horse; blue circles, old; red circles, young; red triangles, FMT donors; blue triangles, FMT recipients.

The fecal microbiota of horses treated with FMT was highly heterogeneous. For 190 pairwise weighted UniFrac distance values between 20 samples (4 samples x 5 recipients), the mean was 0.73 (n = 20x19/2 = 190). This value compares to a mean pairwise distance between healthy horses of 0.44 (n = 2926). The comparison between healthy and diarrheic horses was statistically highly significant (Mann-Whitney Rank Sum test, p<0.001). As apparent in Fig 1, the fecal microbiota of most FMT recipients was very different from that of healthy horses. Clustering of samples from these 2 groups was also statistically significant (ANOSIM R = 0.93 p = 0.001).

Due to the relatively large phylogenetic distances between the microbiota of healthy and of diarrheic horses, the datapoints from healthy horses in Fig 1 appear compressed into a tight cluster. To examine the microbiota of healthy horses for any structure or heterogeneity, the 60 samples originating from healthy animals were analyzed separately. A PCoA colored by geographical origin (S1 Fig) shows a broad overlap between datapoints from different locations. Testing for an effect of location (Bo, RHF, Um and Uc) on the microbiota revealed a significant effect (ANOSIM R 0.29, p<0.001; S6 Table).

**Fig 2:**
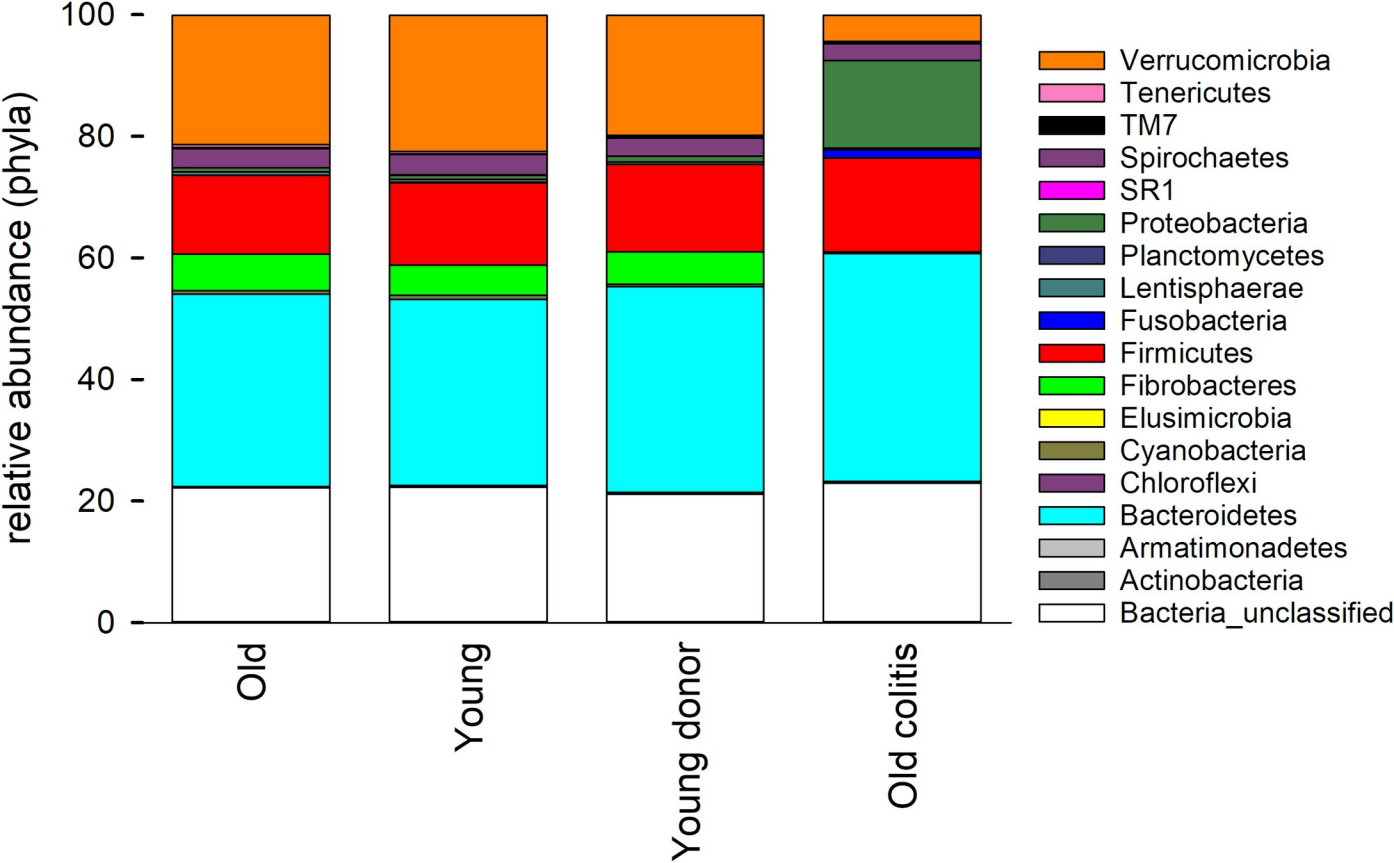
Mean phylum-level phylogeny of the fecal microbiota of healthy and diarrheic horses. Stacked bars represent average relative phyla abundance in the fecal microbiota of 30 healthy old-horse samples, 30 healthy young-horse samples (15 horses collected at two time-points in each age-group), 15 samples from 3 young FMT donor horses (age range: 6–12 years), two technical replicates and 20 samples from 5 geriatric horses with colitis.

Fig 2 shows the mean phylum-level phylogeny for 97 microbiota samples represented in Fig 1 grouped according to age and FMT donor/recipient status. Confirming the data shown in Fig 1, age does not visibly affect the equine fecal microbiota, whereas the presence of diarrhea does. The phylogenetic profile of the donors’ microbiota was broadly representative of that found in healthy animals. Even though the colitis microbiota is diverse, a feature shared by the microbiota of diarrheic horses is a relatively low abundance of Verrucomicrobia. High Proteobacteria abundance was not observed in all animals.

**Fig 3:**
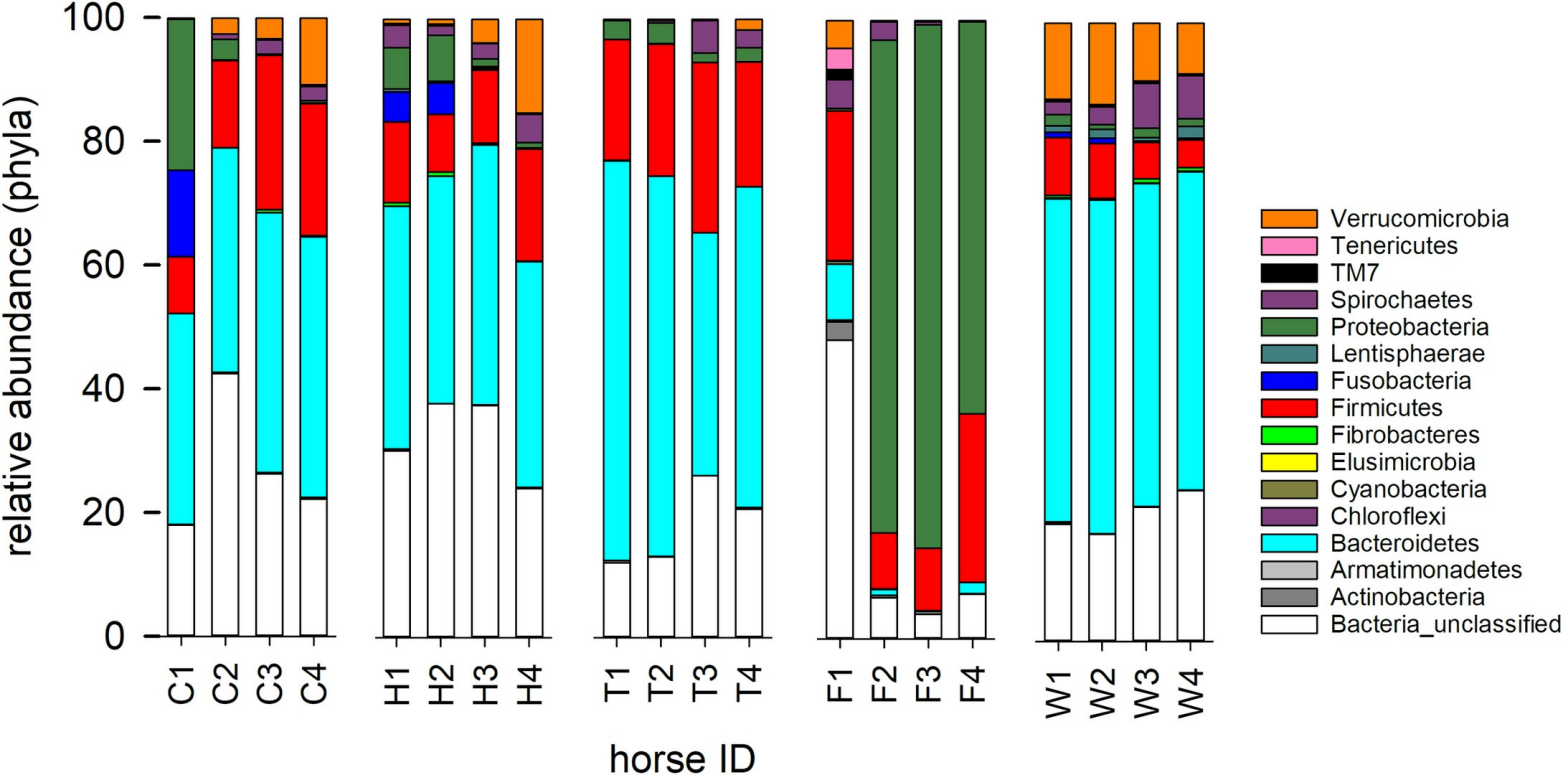
Phylum level taxonomy of 5 FMT recipients. Samples are labelled by horse as shown in S3–S5 Tables. The number indicates the day of treatment.

Classified at phylum level, the microbiota taxonomies (Fig 3) revealed the phylogeny underlying the observed β-diversity among five diarrheic horses apparent in Fig 1. The microbiota of three horses with improving diarrhea scores (Horse C, H and T, S4 Table), which appeared to have responded to treatment and survived to discharge, was characterized by diminishing Proteobacteria abundance and increasing abundance of Verrucomicrobia. There were no apparent commonalities between the profile of the microbiota of the two non-surviving horses (Horse F and W, Fig 3). Microbiota in horse F was dominated by Proteobacteria, specifically by Enterobacteriales. Particularly abundant within this order were sequences classified in the genus *Trabulsiella*. In contrast, W's microbiota was dominated by Bacteroidetes, mostly from the order Bacteroidales. Horse W was ultimately diagnosed with enterolithiasis and Horse F was treated with broad-spectrum antimicrobials throughout the study period (Penicilin G Potassium 22,000 IU/kg intravenously every 6 hours, Pfizerpen, Pfizer, New York, NY; Enrofloxacin 7.5 mg/kg intravenously every 24 hours, Baytril 100, Bayer, Shawnee KS) beginning 4 hours prior to first sampling and FMT. This horse also received one injection of oxytetracycline (7.7 mg/kg intravenously once) from the referring veterinarian the day prior to hospital admission. No other FMT recipients were exposed to systemic or enteral antimicrobials.

**Fig 4:**
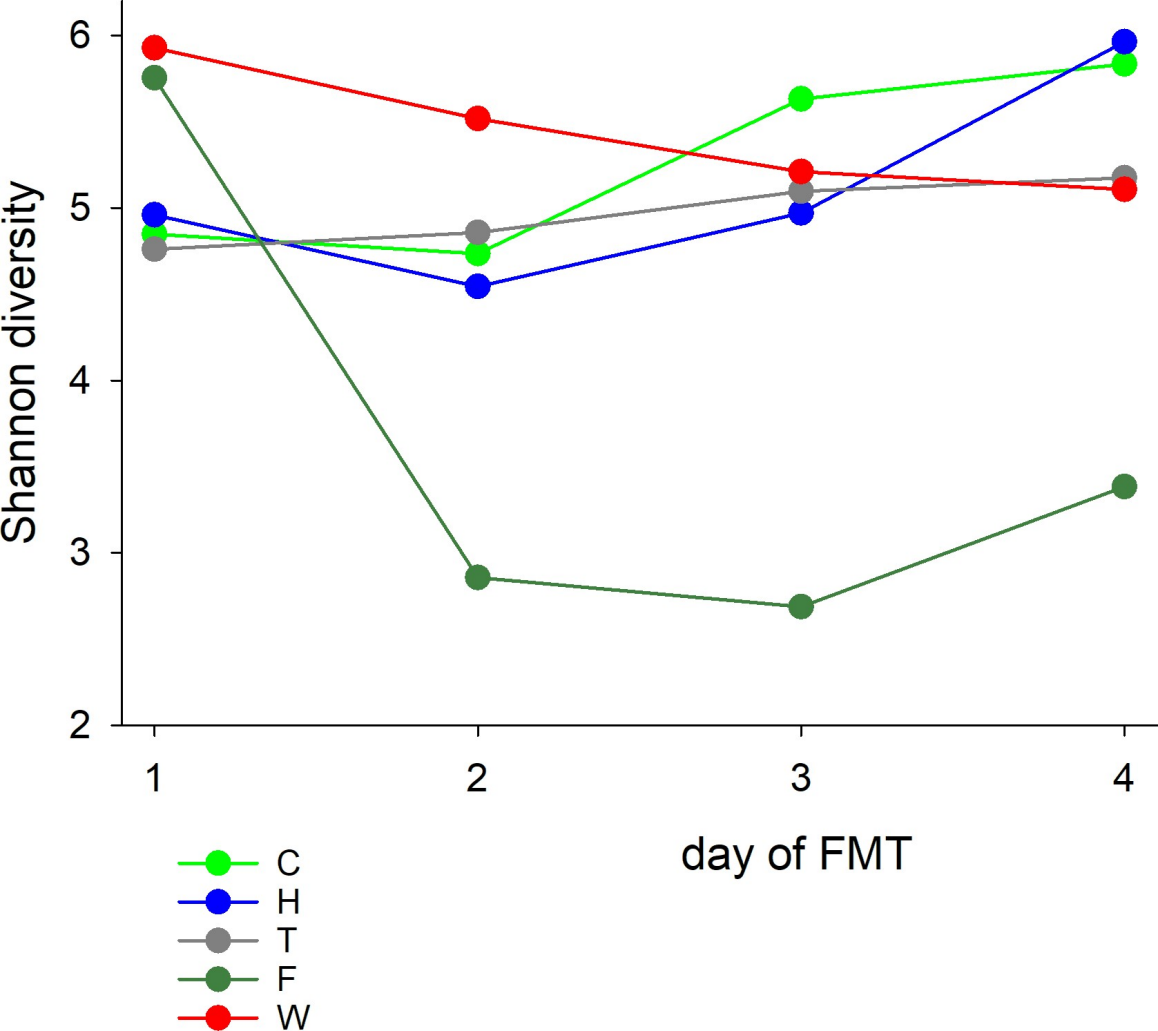
Microbiome α-diversity during FMT treatment. In 3 horses which experienced improving diarrhea scores and survived to discharge (C, H, T), the microbiome became more diverse during the 4-day treatment period. This trend was not observed in horses F and W (non-survivors).

**Fig 5:**
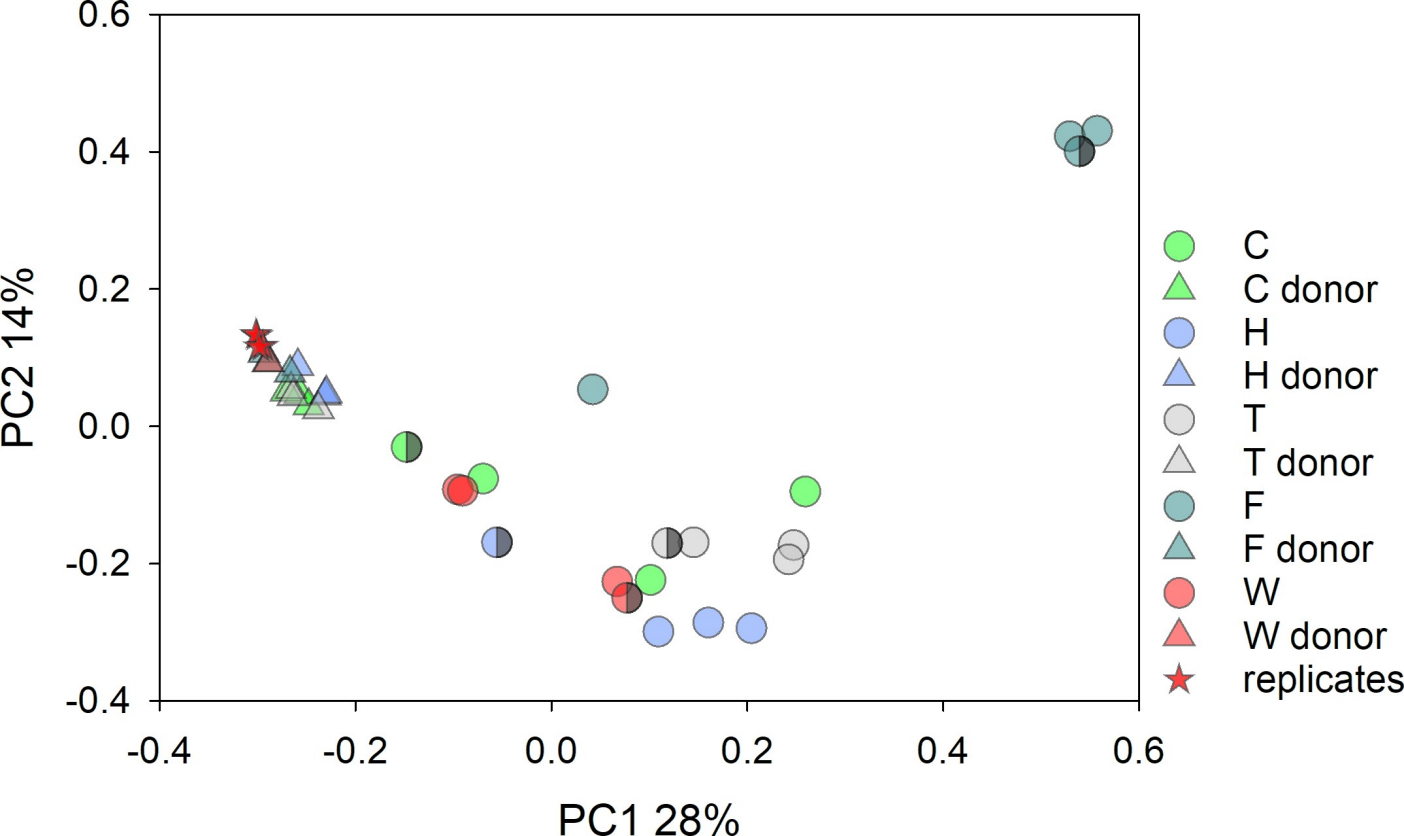
Principal coordinate analysis of FMT donors and recipients. Matched recipient and donor are indicated with the same color. Triangle, donor; circle, recipient. The same color key as in Fig 1 is used. Semi-filled symbol indicates day 4 collection. Stars are technical replicates obtained by processing and sequencing 2 samples from donor horse To twice.

In addition to the increasing Verrucomicrobia abundance mentioned above, two other changes were observed in the 3 horses (C, H, T) that experienced improving diarrhea scores during treatment and survived to discharge: increasing α-diversity and decreasing β-diversity to the donor’s microbiota. The changes in microbiota α-diversity during the 4 FMT days are illustrated in Fig 4. Regardless of the trend towards higher α-diversity in these 3 animals, microbiota diversity in FMT recipients remained low compared to the microbiota diversity in healthy horses. The difference is apparent when comparing the range of Shannon diversity values in Fig 4 and S2 Fig. Decreasing phylogenetic distance between donor and recipient, a characteristic of the microbiota of the 3 surviving horses with diminishing severity of diarrhea, is illustrated in Fig 5. The PcoA shows that the last sample collected on day 4 of FMT (semi-filled circles) resembles more closely the donor (donor A and B) as compared to earlier samples collected on day 1, 2 and 3. Recipients of donor horse To's microbiota (horse F and W) did not show this pattern.

Horses which received FMT from donor horse To (F and W) were transplanted with a very similar microbiome (S3 Fig). The relatively low UniFrac distances between the two FMT groups of 3 samples from donor To (3 transplants given on days 1–3 to two recipients) is apparent when comparing the scale of PCoA axes 1 and 2 in S3 Fig and Fig 1. The different microbiome response of To FMT recipients (horse F vs. W, see Figs 3 and 5) suggests that the gut ecosystem of the recipient played an important role in shaping the microbiome following treatment. More diversity was observed between samples collected from donor A for FMT of horses H and T (S3 Fig). The magnitude of the distance was however relatively small, as can be appreciated by considering the scale of the principal axes.

The relative effect of donor and of day of transplant was further investigated by Canonical Correspondence Analysis using recipient and day, respectively, as independent variables. As apparent in S4 Fig, and inferred from Figs 3 and 5, the transplant recipient had a visible impact on the microbiota. In comparison, individual OTUs profile did not vary as much between the four days of sampling. This is apparent from the large number of OTUs present at similar abundance over time.

**Fig 6:**
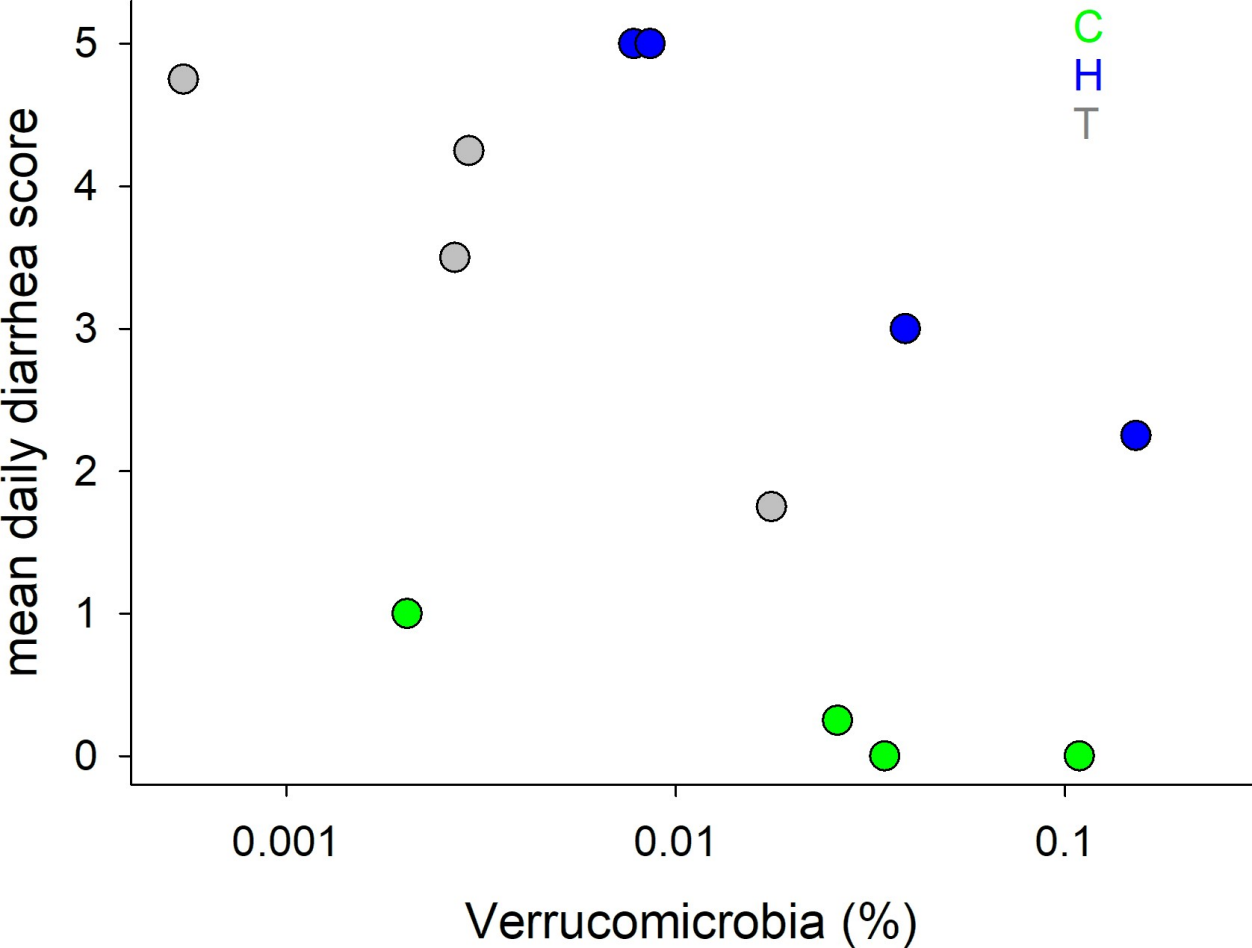
Relative abundance of Verrucomicrobia is negatively correlated with mean daily diarrhea score in transplant recipients. Color indicates FMT recipients as shown in the key.

Fig 3 suggests that Verrucomicrobia abundance increases as diarrhea scores diminish. Changes in the relative abundance of Verrucomicrobia during treatment in the 3 surviving horses which experienced diminishing diarrhea was further analyzed to assess whether these 2 variables are statistically correlated (Fig 6). The correlation between these variables was found to be significant for 2 horses (C and H, p<0.05), but not significant for a third (T; p = 0.063), nor for all datapoints analyzed together (p = 0.06).

## Discussion

Fecal Microbial Transplantation (FMT) was associated with improving diarrhea scores in a small group of surviving horses that presented for acute or progressive diarrhea to a referral center. More specifically, increasing abundance of Verrucomicrobia and increasing α-diversity was observed in treatment responders. The latter group established a microbiome that more closely resembled the donor horse following 3 consecutive daily fecal transplants. In this study, diminishing UniFrac distance over time between donor and FMT recipient was suggestive of transplanted bacteria colonizing the gastro-intestinal tract of the recipient horse and normalizing the intestinal microbiome. Re-establishing a healthy gut microbiome in horses with colitis is considered essential to digestion, is expected to act as a bulwark against pathogenic bacteria and may stimulate the development of a robust and effective immune system to counteract infection. Additionally, normal microbial inhabitants of the gut are thought to help maintain a balance between inflammatory and anti-inflammatory mediators in the intestinal tract,[29] and may stimulate mucus production that prevents the attachment of pathogenic bacteria.[30] The observed increase in relative abundance of mucus-dwelling Verrucomicrobia is consistent with increased mucus production which serves as a barrier to pathogens [31]. As such, FMT could aid in the restoration of gut function in horses with colitis and resolution of diarrhea.

The fecal microbiome of horses that survived to discharge (3/5) showed a significantly higher α-diversity (higher species richness) after FMT treatment and were considered treatment responders. Bacterial species richness and diversity are important elements of a healthy intestinal microbiome, and a reduction in richness and diversity has been associated with conditions such as chronic diarrhea and recurrent *C*. *difficile* infection (CDI) in humans.[32, 33] Moreover, FMT was shown to be highly effective in treating recurrent *Clostridium difficile* infections in people,[34]. While similar data in veterinary species are less abundant to date, FMT has also been associated with faster resolution of parvovirus-associated diarrhea in puppies compared to standard treatments.[35] Therefore, if FMT can establish a more diverse microbiome in horses with colitis, this procedure may represent a cost-effective therapy to facilitate restoration of gut function in horses with dysbiosis or colitis. Anecdotal reports have previously suggested that fecal consistency may normalize following FMT in horses,[36] and that chronic diarrhea may improve in response to microbial transplantation.[9]. A recent study evaluating the efficacy of FMT in the treatment of horses with chronically increased free fecal water showed improved fecal consistency after 14 days post FMT, which persisted throughout the entire study period of 164 days.[37] In contrast to colitis, Free Fecal Water Syndrome (FFWS) is a condition recognized in horses with normal feces that freely pass fecal liquid before, after, or during defecation.[38] Emerging data, therefore, support the potential efficacy of FMT in restoring eubiosis of the gut in horses, similar to observations in people and dogs.

The predominant phyla identified in the fecal microbiome of healthy horses of the current study included Bacteroides and Firmicutes, similar to previous observations by Costa[39] and Morrison;[13] in addition to Verrucomicrobia (see Fig 2). In contrast, a relatively low abundance of Verrucomicrobia was noted in diarrheic horses, with an inverse correlation between diarrhea score and relative Verrucomicrobia abundance. These mucus-dwelling bacteria primarily reside on the intestinal mucosa and are believed to contribute to intestinal health and glucose homeostasis. Previous equine work has demonstrated that horses with colitis may also show decreases in Firmicutes, Actinobacteria, and Spirochetes with an increase in Fusobacteria.[3, 39] The fecal microbiome of most FMT recipients in the current report differed significantly from that of healthy horses, with a high β-diversity between diarrheic horses. For example, there were no apparent commonalities between the microbiome of the 2 non-surviving horses that did not respond to FMT (Horses F and W, Fig 3), indicating a different etiology of colitis. The microbiome of Horse F was extremely rich in Proteobacteria (genus: *Trabulsiella*), a feature that was not observed in the second non-responder (Horse W). Firmicutes were under-represented in W's microbiome and no increase in Verrucomicrobia abundance was observed in this horse following FMT (Fig 3). Horse W was ultimately diagnosed with enterolithiasis and Horse F was treated with broad-spectrum antimicrobials throughout the study period, whereas no other FMT recipients were exposed to systemic or enteral antimicrobials. It has been well established in horses that systemic antimicrobials lead to changes in the intestinal microbiota, with different and specific responses to different antimicrobials.[40] Whether FMT can mitigate or reverse intestinal dysbiosis in the face of concurrent antimicrobial administration has not been established to date.

A recent study at the authors’ institution showed that outcome of colitis was less favorable in geriatric compared to young-adult horses admitted to a referral center, with an 11.8% increase in patient mortality for every year the horse aged.[10] Effective treatment of colitis in geriatric horses thus remains particularly challenging. To optimize FMT protocols in aged horses, the fecal microbiome of healthy geriatric and young-adult horses was compared to determine the utility of geriatric horses as FMT donors.

Geriatric age of donor horses (≥ 20 years old) did not significantly affect the equine fecal microbiome in the population of healthy horses screened in this study. In contrast, previous reports have documented inconsistent effects of advancing age on the equine intestinal microbiome, with both inverse[14] and direct relationships[13] being observed between aging and fecal microbial diversity. The current study suggests that age-matching between donor and FMT recipients is not required to optimize microbial transplantation in adult horses. However, advancing age is generally associated with a higher incidence of age-related health conditions. To reduce the risk of donor comorbidities, individuals aged <60 years are preferred as human fecal donors, based on a recent consensus conference on fecal microbiome transplantation in people.[34] Similar considerations may apply to horses, and reiterate the need to establish standardized protocols to optimize the effects of FMT in clinical practice. For example, choosing donor and recipients that receive similar diets may be more essential than age-matching, to restore intestinal eubiosis in adult horses with colitis.[41]

As expected, we documented that diet affected the fecal microbiome of healthy donor horses. Healthy fecal donor samples were collected from 5 study locations in close geographical proximity. Since different locations also differed by diet, it is not feasible to fully assess the impact of diet by itself on the fecal microbiome. Study location “Uc” was the only site where horses did not have access to pasture. Consistent with a strong effect of diet on the equine fecal microbiome observed by others [14], microbiome samples from “Uc” were significantly different from the other 3 sites in pairwise ANOSIM tests (S6 Table). While access to pasture is reported to increase microbiome diversity, a broad spectrum of factors are known to affect the equine intestinal microbiome, including feed, environmental conditions (e.g. geographic location, climate, hygiene, fasting, transportation, exercise), inflammation, and the use of external compounds, such as prebiotics, probiotics and antibiotics.[39] Our observations support that the effect of diet on the therapeutic impact of FMT should be considered. For example, a greater abundance of Bacillus-Lactobacillus-Streptococcus (BLS) group bacteria was previously reported in horses maintained on a concentrate diet versus a grass-only diet,[42] predicting that variable FMT effects may be based on fecal donor source. Future research aimed at better understanding the association between donor microbiome diversity and effect of FMT should thus be considered. The endpoint of such research would be the identification of a minimal set of bacterial species that could be combined into an optimized synthetic probiotic preparation.[43]

Overall, this preliminary work supports FMT as a mechanism for establishing a more diverse microbiome in horses with colitis and may thus represent a cost-effective therapy to facilitate restoration of gut function in horses with dysbiosis or colitis. As such, surviving horses that responded to FMT with diminishing diarrhea scores in this study were more likely to have improved alpha diversity with increasing abundance of Verrucomicrobia, and acquired a microbiome that began to resemble that of their donor horse. However, larger case-controlled studies are needed to ensure reliability of these results. The fecal microbiome assessment of both healthy geriatric and young-adult horses suggests that age-matching between donor and FMT recipients is not necessary to optimize microbial transplantation in adult horses, while diet-matching may be prudent.

## Supporting information

S1 Table Procedure and sample collection timeline. (DOCX)

S2 Table Healthy age-matched control horse phenotype, diet, and location. (DOCX)

S3 Table Historical information of horses with diarrhea (colitis) receiving FMT. (DOCX)

S4 Table Diarrhea scores (median: range) of horses with colitis throughout the 4-day study period. (DOCX)

S5 Table Clinical parameters (median: range) of horses with colitis throughout the 4-day study period. (DOCX)

S6 Table ANOSIM R values and type I error probability by location. (DOCX)

S1 Fig Equine fecal microbiome by location (Bo, RHF, Uc, Um) and age (young vs. aged). Black, Location Bo; grey, Location RHF; brown, Location Uc; turquoise, Location Um. Circles, aged healthy horses; diamonds, young-adults.
(TIFF)

S2 Fig Fecal microbiome α-diversity in healthy horses by age. (TIFF)

S3 Fig Principal coordinate analysis of donor samples used in FMT. Color indicates recipient as used in Figs 4, 5 and 6. Symbols indicate the time-point of sample collection. Green, Horse C; blue, Horse H; grey, Horse T; turquoise, Horse F; red, Horse W; star, replicates; Half-shaded shapes indicate the last sample collected from recipients.
(TIFF)

S4 Fig Canonical Correspondence Analysis biplots illustrating OTU distribution among 3 FMT recipients and among 4 days of transplant. **Left**. FMT recipients are represented with red triangles, labelled as in Figs 3–5). The distance between triangles approximates the average dissimilarity between their OTU composition, i.e., their bacterial microbiota taxonomy. To maximize clarity, only 80 out of 321 OTUs are displayed. The distance between pie symbols approximates the dissimilarity between the relative abundance of OTUs across fecal samples, such that overlapping OTUs or OTUs in close proximity to each other tend to occur together (in the same samples). Segmentation of pie symbols represents the OTU’s relative abundance in samples collected from each horse, as indicated by the same colors as in Figs 4 and 5. For instance, a pie that is 100% blue represents an OTU that only occurred in FMT recipient H. The OTU distribution pattern shows that the 3 recipients, in spite of showing similar microbiota patterns (like increasing alpha-diversity over time, Fig 4) were populated by very different microbiota. **Right**. FMT days are represented with red triangles. Each pie symbol represents an OTU. Pie segmentation represents the OTU’s relative abundance in samples collected on each FMT day, as indicated by white-to-black shading, where white is day 1, light grey day 2, dark grey day 3, and black day 4. The distances between a pie symbol and each triangle shows the relative abundance of that OTU on individual FMT days. In other words, the OTU is predicted to be most abundant in the FMT day located at the shortest distance on the plot. For additional explanations, see left panel legend.
(TIFF)
