# Supplementary material for: The fecal microbiota of healthy donor horses and geriatric recipients undergoing fecal microbial transplantation for the treatment of diarrhea
Source: PLoS One. 2020 Mar 10;15(3):e0230148. doi: 10.1371/journal.pone.0230148 (PMC7064224; doi:10.1371/journal.pone.0230148)
Supplement: S1 Table — (DOCX) [file pone.0230148.s001.docx]

**Table S1:** Procedure and Sample Collection Timeline

| **Animal Group** | **Days** | **1** | **2** | **3** | **4** |
| --- | --- | --- | --- | --- | --- |
| **Colitis Group (n = 5)** | FMT  Physical exam  Feces collection  16S amplicon sequencing | x  x  x  x | x  x  x  x | x  x  x  x | x  x  x |
| **Healthy Donor Horses (n = 3)** | Feces collection  16S amplicon sequencing | x  x | x  x | x  x |  |
