# Supplementary material for: The fecal microbiota of healthy donor horses and geriatric recipients undergoing fecal microbial transplantation for the treatment of diarrhea
Source: PLoS One. 2020 Mar 10;15(3):e0230148. doi: 10.1371/journal.pone.0230148 (PMC7064224; doi:10.1371/journal.pone.0230148)
Supplement: S2 Table — (DOCX) [file pone.0230148.s002.docx]

**Table S2:** Healthy Age-Matched Control Horse phenotype, diet, and location

| **Horse Number** | **Age** (years) | **Gender** | **BW** (kg) | **BCS** (1-9) | **Hay** (type and percentage of body weight per day) | **Concentrate Type** | **Concentrate** (lb per day) | **Pasture**  **turn- out** (hours/day) | **Location** |
| --- | --- | --- | --- | --- | --- | --- | --- | --- | --- |
| 1 | 10 | Mare | 567 | 8 | 2% BW 2^nd^ Cut Grass Hay | 30% Protein, 3% Fat pellets | 1 | 6 | CM |
| 2 | 23 | Mare | 582 | 8.5 | 2% BW 2^nd^ Cut Grass Hay | 30% Protein, 3% Fat pellets | 1 | 6 | CM |
| 3 | 5 | Mare | 395 | 8 | 2% BW 1^st^ Cut Grass Hay | 30% Protein, 3% Fat pellets | 1 | 6 | CM |
| 4 | 23 | Mare | 630 | 8.5 | 2% BW 1^st^ Cut Grass Hay | 30% Protein, 3% Fat pellets | 1 | 6 | CM |
| 5 | 10 | Mare | 480 | 8 | 2% BW 1^st^ Cut Grass Hay | 30% Protein, 3% Fat pellets | 1 | 6 | CM |
| 6 | 22 | Mare | 513 | 9 | 2% BW 1^st^ Cut Grass Hay | 30% Protein, 3% Fat pellets | 1 | 6 | CM |
| 7 | 7 | Gelding | 542 | 6 | 2% BW 1^st^ Cut Grass Hay | 14% Protein, 10% Fat pellets | 3.75 | 24 | CM |
| 8 | 23 | Gelding | 526 | 5 | 2% BW 1^st^ Cut Grass Hay | 14% Protein, 10% Fat pellets | 3.75 | 24 | CM |
| 9 | 2 | Mare | 384 | 6 | 2- 2.5% BW 1^st^ Cut Grass Hay | 13% Protein, 8% Fat pellets | 6 | 0 | ECC |
| 10 | 20 | Mare | 421 | 5 | 2- 2.5% BW 1^st^ Cut Grass Hay | 13% Protein, 8% Fat pellets | 4 | 0 | ECC |
| 11 | 2 | Mare | 415 | 7 | 2- 2.5% BW 1^st^ Cut Grass Hay | 13% Protein, 8% Fat pellets | 6 | 0 | ECC |
| 12 | 22 | Mare | 526 | 6 | 2- 2.5% BW 1^st^ Cut Grass Hay | 13% Protein, 8% Fat pellets | 8 | 0 | ECC |
| 13 | 7 | Mare | 225 | 7 | 2- 2.5% BW 1^st^ Cut Grass Hay | 13% Protein, 8% Fat pellets | 6 | 0 | ECC |
| 14 | 25 | Mare | 493 | 7 | 2- 2.5% BW 1^st^ Cut Grass Hay | 13% Protein, 8% Fat pellets | 6 | 0 | ECC |
| 15 | 2 | Mare | 405 | 7 | 2- 2.5% BW 1^st^ Cut Grass Hay | 13% Protein, 8% Fat pellets | 6 | 0 | ECC |
| 16 | 20 | Mare | 487 | 8 | 2- 2.5% BW 1^st^ Cut Grass Hay | 13% Protein, 8% Fat pellets | 6 | 0 | ECC |
| 17 | 2 | Mare | 443 | 5 | 2- 2.5% BW 1^st^ Cut Grass Hay | 13% Protein, 8% Fat pellets | 6 | 0 | ECC |
| 18 | 23 | Mare | 509 | 5 | 2- 2.5% BW 1^st^ Cut Grass Hay | 13% Protein, 8% Fat pellets | 10 | 0 | ECC |
| 19 | 10 | Mare | 539 | 5 | 2- 2.5% BW 1^st^ Cut Grass Hay | 13% Protein, 8% Fat pellets | 10 | 0 | ECC |
| 20 | 21 | Mare | 456 | 4.5 | 2- 2.5% BW 1^st^ Cut Grass Hay | 13% Protein, 8% Fat pellets | 8 | 0 | ECC |
| 21 | 4 | Gelding | 442 | 5 | 2- 2.5% BW 1^st^ Cut Grass Hay | 13% Protein, 8% Fat pellets | 6 | 0 | ECC |
| 22 | 24 | Mare | 441 | 4 | 2- 2.5% BW 1^st^ Cut Grass Hay | 13% Protein, 8% Fat pellets | 6 | 0 | ECC |
| 23 | 6 | Gelding | 504 | 7 | 2% BW 1^st^ Cut Grass Hay | 30% Protein, 5% Fat pellets | 1.25 | 14 | SECM |
| 24 | 22 | Gelding | 510 | 7 | 2% BW 1^st^ Cut Grass hay | 30% Protein, 5% Fat pellets | 1.25 | 14 | SECM |
| 25 | 11 | Gelding | 663 | 6.5 | 1-1.5% BW 2^nd^ Cut Grass Hay | 14% Protein, 7% Fat pellets | 4 | 3 | SCM |
| 26 | 20 | Gelding | 432 | 5 | 1% BW 2^nd^ Cut Grass Hay | 14% Protein, 7% Fat pellets | 3 | 6 | SCM |
| 27 | 12 | Mare | 523 | 5.5 | 1-1.5% BW 2^nd^ Cut Grass Hay | 14% Protein, 7% Fat pellets | 3 | 3 | SCM |
| 28 | 25.5 | Mare | 505 | 4 | 1% BW 2^nd^ Cut Grass Hay | 14% Protein, 7% Fat pellets | 3 | 6 | SCM |
| 29 | 10 | Gelding | 574 | 6 | 1 -1.5% BW 2^nd^ Cut Grass Hay | 14% Protein, 7% Fat pellets | 4 | 3 | SCM |
| 30 | 25 | Gelding | 564 | 6 | 1% BW 2^nd^ Cut Grass Hay | 14% Protein, 7% Fat pellets | 3 | 6 | SCM |

**BCS**, Body Condition Score: 1 = Emaciated, 4 = Ideal, 9 = Obese; **BW**, Body Weight. **CM,** Central Massachusetts; **ECC,** East Central Connecticut; **SECM,** South East Central Massachusetts; **SCM,** South Central Massachusetts; Highlighted rows denote young-adult (2-12 years old) vs. geriatric (≥20 years old) horse pairings to evaluate the effect of age on fecal microbiome.
