# Supplementary material for: The fecal microbiota of healthy donor horses and geriatric recipients undergoing fecal microbial transplantation for the treatment of diarrhea
Source: PLoS One. 2020 Mar 10;15(3):e0230148. doi: 10.1371/journal.pone.0230148 (PMC7064224; doi:10.1371/journal.pone.0230148)
Supplement: S3 Table — (DOCX) [file pone.0230148.s003.docx]

**Table S3:** Historical Information of horses with diarrhea (colitis) receiving FMT

| **Patient ID** | **Age**  (years) | **Breed** | **Gender** | **BCS** (1-9) | **Presenting Complaint** (outside of diarrhea) | **Duration of diarrhea prior to first FMT** | **Duration of diarrhea following start of FMT** |
| --- | --- | --- | --- | --- | --- | --- | --- |
| C | 22 | Paint | Gelding | 6 | Colic, Elevated Creatinine | 24 hours | 11 hours |
| H | 25.5 | Welsh Pony | Mare | 4 | Colic | > 5 years | 6.1 days |
| T | 21 | Thoroughbred | Gelding | 6 | Fever, Inappetence, Colic | 24 hours | 3.7 days |
| F | 29 | Morgan | Gelding | 5 | Fever, Nasal discharge | 6 hours | 3 days |
| W | 20 | Paint Pony | Mare | 7 | Colic | 2 years | 6.75 days |

**Body Condition Score**, BCS: 1 = Emaciated, 4 = Ideal, 9 = Obese
