# Supplementary material for: The fecal microbiota of healthy donor horses and geriatric recipients undergoing fecal microbial transplantation for the treatment of diarrhea
Source: PLoS One. 2020 Mar 10;15(3):e0230148. doi: 10.1371/journal.pone.0230148 (PMC7064224; doi:10.1371/journal.pone.0230148)
Supplement: S4 Table — (DOCX) [file pone.0230148.s004.docx]

**Table S4:** Diarrhea Scores (median: range) of horses with colitis throughout the 4-day study period

| **Patient ID** | **FMT donor** | **Manure at Enrollment** | **Day 1:** Median Manure Score (range) | **Day 2:** Median Manure Score (range) | **Day 3:** Median Manure Score (range) | **Day 4:** Final Manure Score | **Outcome** |
| --- | --- | --- | --- | --- | --- | --- | --- |
| C | Donor B | 5 | 0.5 (0-2) | 0.25 (0 - 1) | 0 (0) | 0 | Survived |
| H | Donor A | 5 | 5 (5) | 5 (5) | 3 (3) | 2 | Survived |
| T | Donor A | 5 | 4.75 (4-5) | 4.25 (4 – 5) | 3.5 (3-4) | 0 | Survived |
| F | Donor To | 3 | 3.5 (3-4) | No manure observed | No manure observed | 0 | Euthanized |
| W | Donor To | 5 | 5 (5) | 5 (5) | 5 (5) | 5 | Euthanized |

**Manure Scoring System:** 0: Normal, firm but moist balls of manure, 1: Soft-formed balls of manure that lose their form upon reaching the ground, 2: Pudding-consistency manure that still holds some shape, 3: Pudding-consistency manure that spreads out upon reaching the ground, 4: Watery manure with some formed pieces, 5: Water manure without formed pieces.
