# Supplementary material for: The fecal microbiota of healthy donor horses and geriatric recipients undergoing fecal microbial transplantation for the treatment of diarrhea
Source: PLoS One. 2020 Mar 10;15(3):e0230148. doi: 10.1371/journal.pone.0230148 (PMC7064224; doi:10.1371/journal.pone.0230148)
Supplement: S5 Table — (DOCX) [file pone.0230148.s005.docx]

**Table S5:** Clinical Parameters (median: range) of horses with colitis throughout the 4-day study period

| **Patient ID** | **Heart Rate**  bpm | **Respiratory rate** brpm | **Temperature**  oF | **Mucous Membranes** | **Packed Cell Volume** % | **Total Solids** g/dL | **Serum Lactate** mmol/L |
| --- | --- | --- | --- | --- | --- | --- | --- |
| C | 40 (36 - 44) | 16 (16 - 20) | 99.3 (98.9 - 99.8) | Normal | 39 (36 - 45) | 7.1 (6.6 - 7.2) | 1.0 (1.0) |
| H | 36 (32 - 36) | 12 (12) | 100.2 (99.8 - 100.6) | Normal | 38 (36 - 39) | 7.0 (6.7 - 7.9) | -- |
| T | 34 (32 - 40) | 14 (12 - 16) | 99.8 (99.5 - 100.5) | Normal | 33 (29 - 36) | 7.3 (6.6 - 7.4) | 0.5 (0.4 - 0.6) |
| F | 46 (44 - 52) | 16 (16 - 24) | 100.3 (99.8 - 101.4) | Normal | 28 (25 - 30) | 6.4 (5.3 - 7.0) | 1.4 (0.9 - 1.9) |
| W | 50 (44 - 52) | 18 (16 - 20) | 100.5 (100.3 - 102.1) | Normal | 37 (36 - 40) | 6.3 (6.0 - 7.2) | 0.7 (0.6 - 0.9) |

**Heart rate**, bpm: beats per minute; **Respiratory rate**, brpm: breaths per minute; **Mucous membranes**, normal infers: light pink, moist, capillary refill time < 2 seconds
