# Supplementary material for: The fecal microbiota of healthy donor horses and geriatric recipients undergoing fecal microbial transplantation for the treatment of diarrhea
Source: PLoS One. 2020 Mar 10;15(3):e0230148. doi: 10.1371/journal.pone.0230148 (PMC7064224; doi:10.1371/journal.pone.0230148)
Supplement: S6 Table — (DOCX) [file pone.0230148.s006.docx]

**Table S6:** ANOSIM R values and type I error probability by location

| **Comparison** | **R-value** | **P-value** |
| --- | --- | --- |
| Bo-RHF-Um-Uc | 0.286 | **<0.001** |
| Bo-RHF | 0.064 | 0.265 |
| Bo-Um | 0.230 | 0.101 |
| Bo-Uc | 0.366 | **0.018** |
| RHF-Um | 0.342 | **<0.001** |
| RHF-Uc | 0.376 | **<0.001** |
| Um-Uc | 0.185 | **<0.001** |

Bolted P-values represent statistically significant differences between locations.
