# Supplementary figures and images for: The fecal microbiota of healthy donor horses and geriatric recipients undergoing fecal microbial transplantation for the treatment of diarrhea
Source: PLoS One. 2020 Mar 10;15(3):e0230148. doi: 10.1371/journal.pone.0230148 (PMC7064224; doi:10.1371/journal.pone.0230148)

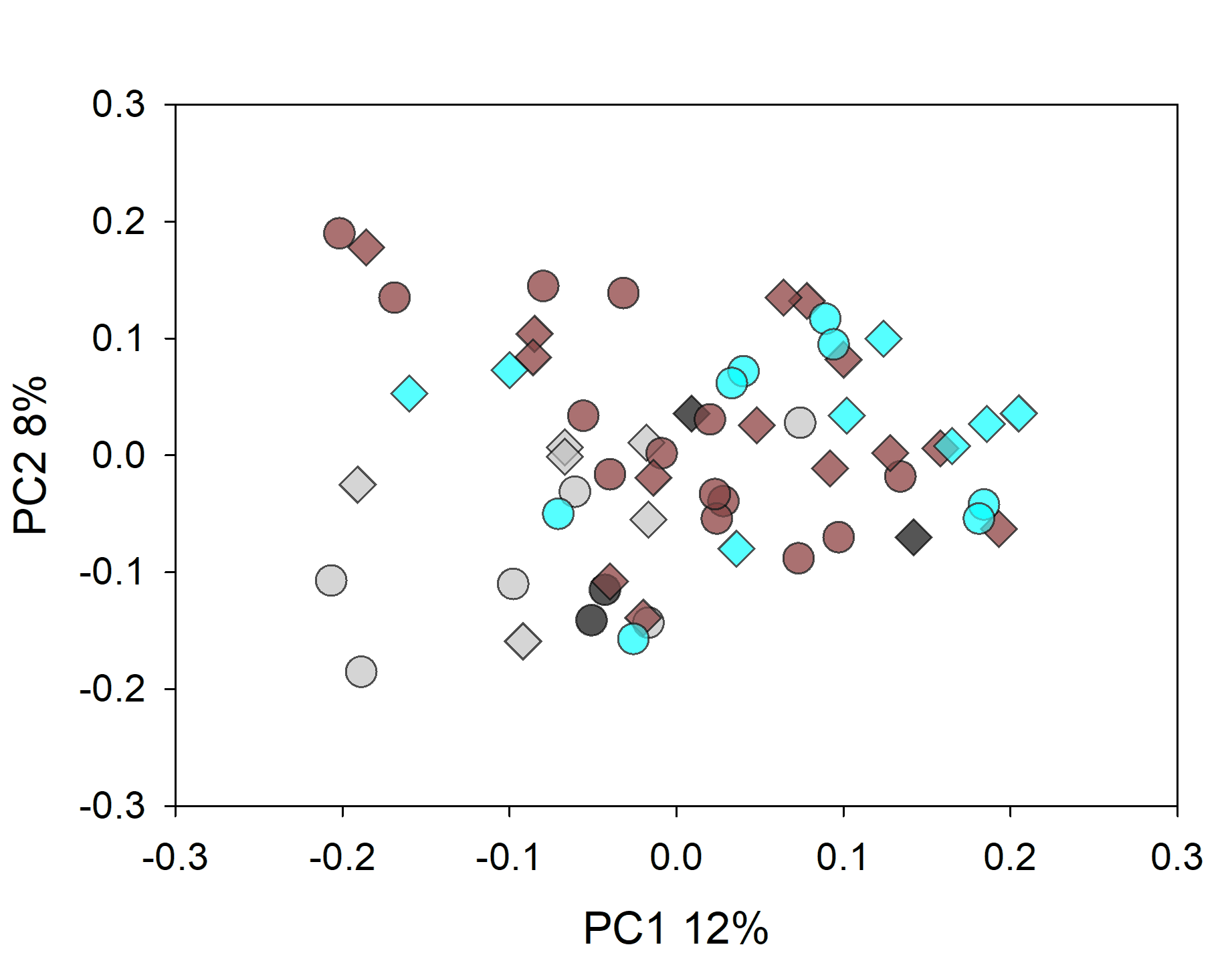

Supplement: S1 Fig — Black, Location Bo; grey, Location RHF; brown, Location Uc; turquoise, Location Um. Circles, aged healthy horses; diamonds, young-adults. (TIFF) [file pone.0230148.s007.tiff]

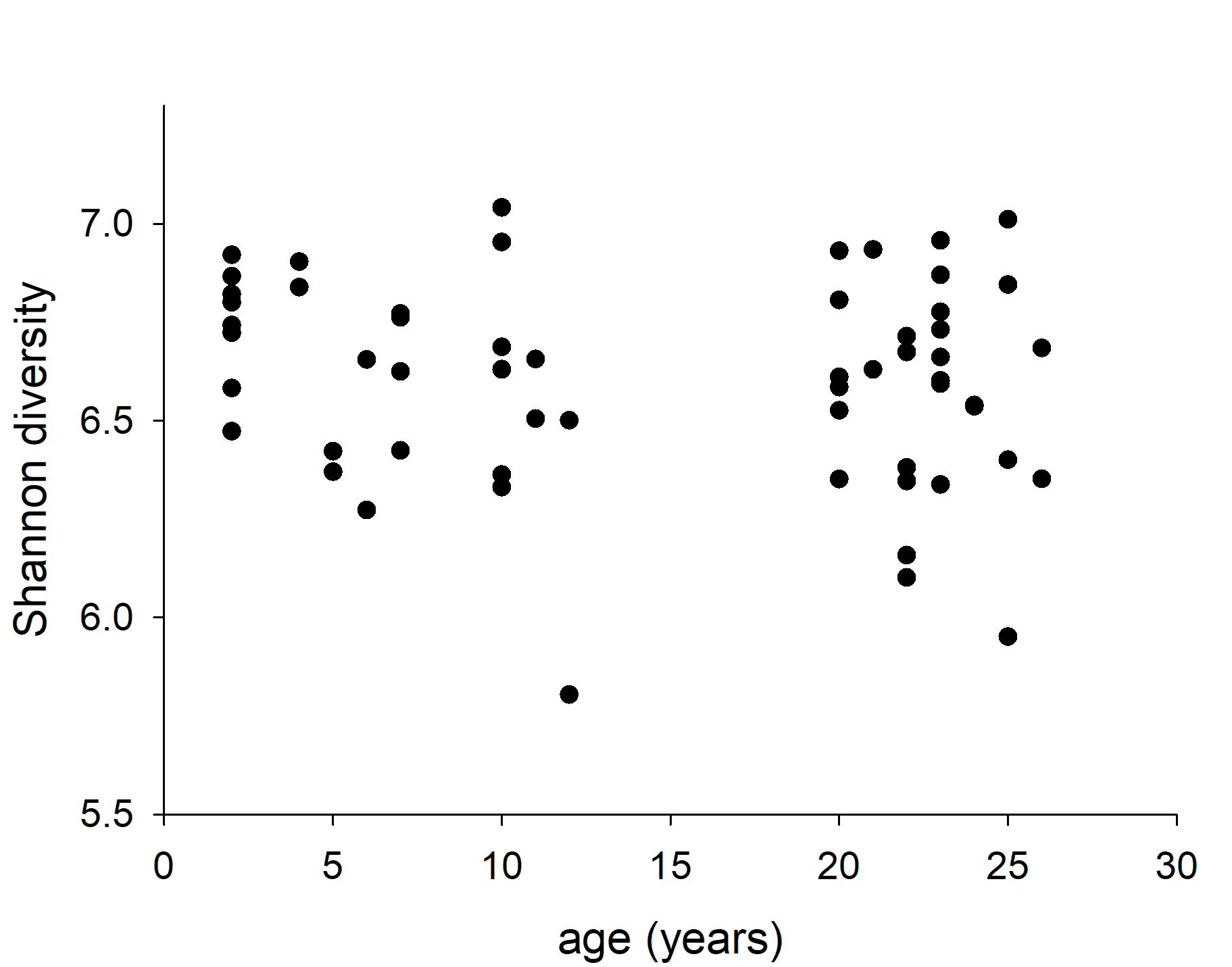

Supplement: S2 Fig — (TIFF) [file pone.0230148.s008.tiff]

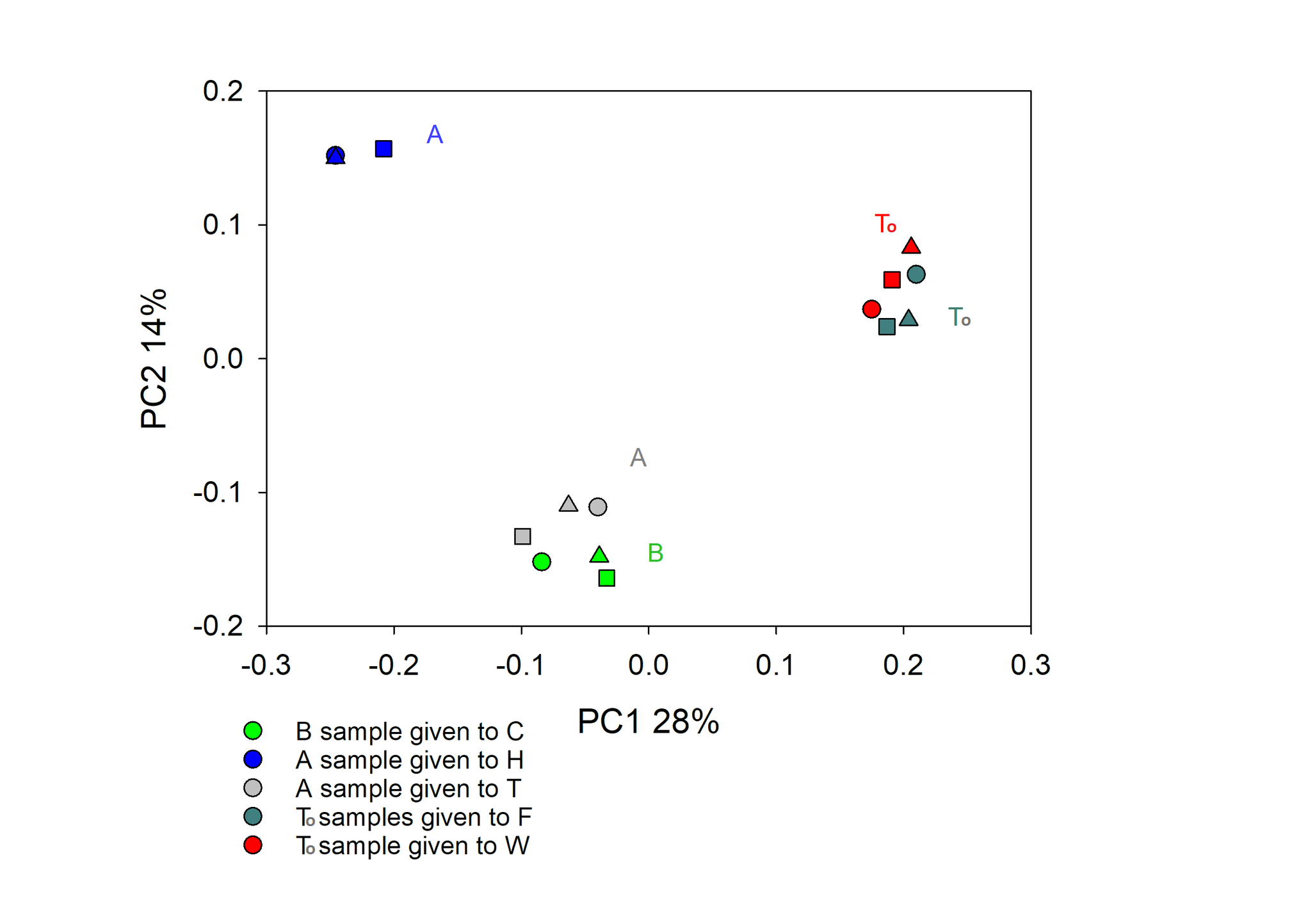

Supplement: S3 Fig — Color indicates recipient as used in Figs 4, 5 and 6. Symbols indicate the time-point of sample collection. Green, Horse C; blue, Horse H; grey, Horse T; turquoise, Horse F; red, Horse W; star, replicates; Half-shaded shapes indicate the last sample collected from recipients. (TIFF) [file pone.0230148.s009.tiff]

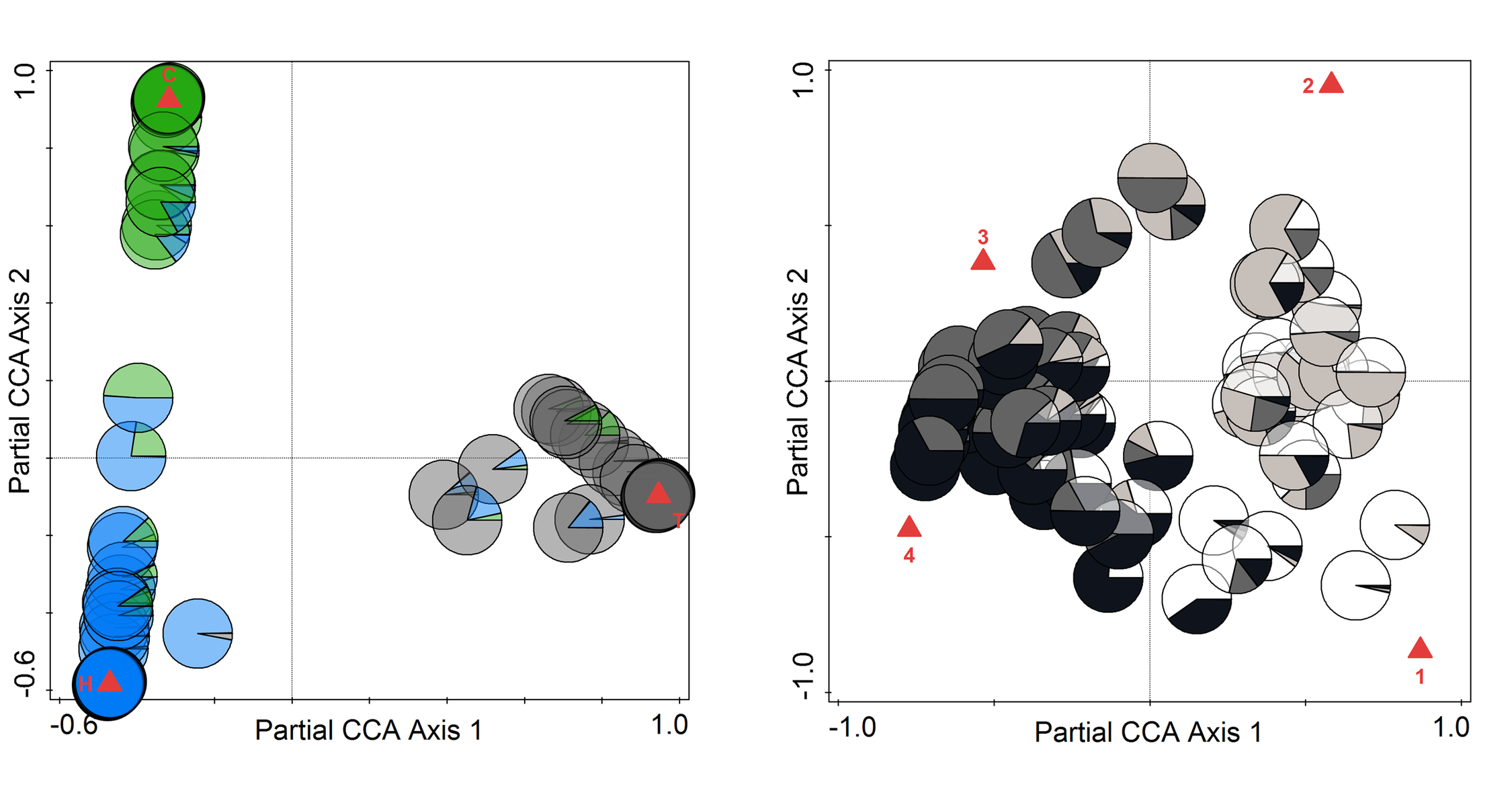

Supplement: S4 Fig — Left. FMT recipients are represented with red triangles, labelled as in Figs 3–5). The distance between triangles approximates the average dissimilarity between their OTU composition, i.e., their bacterial microbiota taxonomy. To maximize clarity, only 80 out of 321 OTUs are displayed. The distance between pie symbols approximates the dissimilarity between the relative abundance of OTUs across fecal samples, such that overlapping OTUs or OTUs in close proximity to each other tend to occur together (in the same samples). Segmentation of pie symbols represents the OTU’s relative abundance in samples collected from each horse, as indicated by the same colors as in Figs 4 and 5. For instance, a pie that is 100% blue represents an OTU that only occurred in FMT recipient H. The OTU distribution pattern shows that the 3 recipients, in spite of showing similar microbiota patterns (like increasing alpha-diversity over time, Fig 4) were populated by very different microbiota. Right. FMT days are represented with red triangles. Each pie symbol represents an OTU. Pie segmentation represents the OTU’s relative abundance in samples collected on each FMT day, as indicated by white-to-black shading, where white is day 1, light grey day 2, dark grey day 3, and black day 4. The distances between a pie symbol and each triangle shows the relative abundance of that OTU on individual FMT days. In other words, the OTU is predicted to be most abundant in the FMT day located at the shortest distance on the plot. For additional explanations, see left panel legend. (TIFF) [file pone.0230148.s010.tiff]
